# Dietary Exposures and Interventions in Inflammatory Bowel Disease: Current Evidence and Emerging Concepts

**DOI:** 10.3390/nu15030579

**Published:** 2023-01-22

**Authors:** John Gubatan, Chiraag V. Kulkarni, Sarah Melissa Talamantes, Michelle Temby, Touran Fardeen, Sidhartha R. Sinha

**Affiliations:** 1 Division of Gastroenterology and Hepatology, Stanford University School of Medicine, Stanford, CA 94305, USA; 2 Chan Zuckerberg Biohub, San Francisco, CA 94158, USA

**Keywords:** food, diet, inflammatory bowel disease, ulcerative colitis, Crohn’s disease, clinical trials, epidemiology

## Abstract

Diet is intimately linked to the gastrointestinal (GI) tract and has potent effects on intestinal immune homeostasis. Inflammatory bowel disease (IBD) is characterized by chronic inflammation of the GI tract. The therapeutic implications of diet in patients with IBD have received significant attention in recent years. In this review, we provide a contemporary and comprehensive overview of dietary exposures and interventions in IBD. Epidemiological studies suggest that ultra-processed foods, food additives, and emulsifiers are associated with a higher incidence of IBD. Exclusion and elimination diets are associated with improved symptoms in patients with IBD, but no effects on objective markers of inflammation. Specific dietary interventions (e.g., Mediterranean, specific carbohydrate, high fiber, ketogenic, anti-inflammatory diets) have been shown to reduce symptoms, improve inflammatory biomarkers, and quality of life metrics to varying degrees, but these studies are limited by study design, underpowering, heterogeneity, and confounding. To date, there is no robust evidence that any dietary intervention alone may replace standard therapies in patients with IBD. However, diet may play an adjunct role to induce or maintain clinical remission with standard IBD therapies. The results of novel dietary trials in IBD such as personalized fiber, intermittent fasting, and time-restricted diets are eagerly awaited.

## 1. Introduction

Inflammatory bowel disease (IBD), which includes Crohn’s disease (CD) and ulcerative colitis (UC), is a chronic inflammatory disorder of the gastrointestinal tract. The incidence of IBD is increasing worldwide in both industrialized and developing countries [1]. Although genetic predisposition is a major factor in the pathogenesis of IBD, environmental exposures are being increasingly recognized to contribute to the risk of developing IBD. Indeed, the incidence in IBD has paralleled changes in diet, lifestyle, and industrialization [2]. Currently, the mainstay of therapy in IBD involves immunosuppressive drugs such as steroids, immunomodulators, biologics, and small molecule inhibitors to curb gastrointestinal inflammation [3]. However, some of these pharmacologic therapies carry a risk of infection, malignancy, and adverse reactions. There is an unmet clinical need for non-pharmacologic strategies to manage IBD. Given that diet is intimately linked with the gastrointestinal tract and that nutritional deficiencies are common in IBD [2], there has been significant interest in exploring dietary exposures as modifiable risk factors in IBD and dietary interventions as direct or adjunct therapies in patients with IBD. In this review, we provide a contemporary and comprehensive overview of the role of dietary exposures and interventions in IBD. First, we review the association and potential mechanisms of ultra-processed foods and food additives contributing to the risk of IBD in both animal and human studies. Second, we review the impact of elimination and exclusion diets (e.g., low FODMAP, lactose-free, gluten-free, carrageenan-free, low microparticle diets) and clinical outcomes in IBD. Third, we review specific dietary interventions (e.g., Mediterranean, specific carbohydrate, ketogenic, plant-based, anti-inflammatory diets, etc.) from both clinical trials and cohort studies in IBD. Finally, we highlight emerging concepts and ongoing dietary clinical trials in IBD as well as challenges and future directions in this field. Table 1 provides a summary of dietary patterns and their descriptions included in this review. Table S1 (Supplementary Materials) provides a succinct summary of the included clinical dietary studies, study design, patient population, and clinical outcomes. 

## 2. Food Processing and Additives and Risk of IBD

### 2.1. Ultra-Processed Foods in Inflammatory Bowel Disease

**Figure 1:**
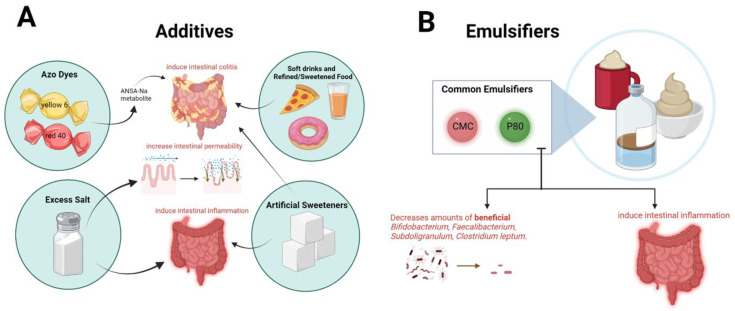
Ultra-processed Foods and Food Additives in IBD. (**A**) Additives and their effects on IBD: Excessive salt and artificial sweeteners can promote intestinal inflammation and induce colitis. Higher salt concentrations have been shown to increase intestinal permeability, increase inflammatory cytokine production through a reduction in fecal short-chain fatty acid production and depletion of Lactobacillus, and exacerbate chemically induced colitis in experimental models. Artificial sweeteners in UPFs may also induce gut inflammation, as seen in mice models of spontaneous ileitis with sucralose/maltodextrin supplementation. Azo dyes red 40 and yellow 6, the most abundant synthetic food coloring used by the food industry, can trigger IBD-like colitis in genetically susceptible mice. Consumption of ≥3 servings/week of soft drinks, consumption of ≥100 g/day of refined, sweetened foods, and consumption of ≥100 g/day of salty snacks were all associated with a higher risk of IBD. (**B**) Common Emulsifiers P80 and CMC effects on Gut and Microbiome: P80 and CMC are the most studied emulsifiers. They have been found to cause similar alterations to human gut microbiomes as does IBD. CMC and P80 are found in edible oils, ice creams, cake mixes, icing, and chocolate syrup, but ingestion of them has led to reduced numbers of beneficial Bifidobacterium and important SCFA producers—Faecalibacterium and Subdoligranulum—and Clostridium leptum. These same microbiota alterations by P80 and CMC in mice led to chronic intestinal inflammation.

Consumption of highly processed foods has occurred in developing and developed countries at a rapid rate, just as the incidence of chronic inflammatory diseases, such as IBD, has increased dramatically [4]. This correlation has led many to question whether a link exists between food processing and the risk of chronic diseases such as CD and UC (Figure 1). The 2021 International Prospective Urban Rural Epidemiology (PURE) cohort [5] presented data showing a significant association between ultra-processed food (UPF) consumption and an increased risk of CD. UPF consumption also showed an association with an increased risk of UC, although the relationship was not as significant.

Processed food additives, such as azo dyes red 40 and yellow 6, the most abundant synthetic food coloring used by the food industry, can trigger IBD-like colitis in genetically susceptible mice [4]. Commensal bacteria, i.e., Bacteroides ovatus and Enterococcus faecalis, can metabolize these food dyes to produce a metabolite known as 1-amino-2-naphthol-6-sulphonate sodium salt (ANSA-Na) which appears to induce colitis [4]. This is important to note since intestinal microbiota seem to play a key role in determining the harmful effects of food additives on intestinal health.

Ultra-processed food consumption may influence the development of IBD through various mechanisms. One hypothesis is that higher UPF consumption may be associated with the replacement of “unprocessed or minimally processed foods (UMPs)” that typically contain abundant amounts of fiber [6]. Another hypothesis is that ultra-processed foods with additives, such as excessive salt and artificial sweeteners, can promote intestinal inflammation. Higher salt concentrations have been shown to increase intestinal permeability, increase inflammatory cytokine production through a reduction in fecal short-chain fatty acid production and depletion of Lactobacillus, and exacerbate chemically induced colitis in experimental models [6]. Artificial sweeteners in UPFs may also induce gut inflammation, as seen in mice models of spontaneous ileitis with sucralose/maltodextrin supplementation; inflammatory bacteria, such as Salmonella, that flourish under sucralose/maltodextrins supplementation, affect gut epithelial cells by reducing mucus production, and enhancing colitis susceptibility [6].

In the global prospective cohort study by Narula et al. [7], higher intake of ultra-processed food was associated with a higher risk of incident IBD, especially in participants consuming ≥5 servings/day. Data from the study also demonstrated that higher intake (≥5 servings/day vs. <1 serving per day) of processed meat was associated with a greater risk of IBD (hazard ratio (hazard ratio 1.82, 95% confidence interval 1.22–2.72, *p* = 0.006). The highest intake of soft drinks (≥3 servings/week) compared to the lowest intake (<0.5 serving/week) was associated with a higher risk of IBD. Consumption of ≥3 servings/week of soft drinks, consumption of ≥100 g/day of refined, sweetened foods, and consumption of ≥100 g/day of salty snacks were all associated with a higher risk of IBD. Intake of just one serving of fried food per day showed the highest risk of IBD compared to those consuming zero servings per day. In summary, a higher intake of ultra-processed food was associated with higher risk of IBD [7].

In a European prospective cohort study that followed CD and UC patients for 13 years, 179 incident cases of CD and 431 incident cases of UC were identified. Risk of Crohn’s was lower in people consuming high proportions of unprocessed or minimally processed foods; hazard ratios were adjusted for the highest vs. lowest quartile: 0.57 (95% CI: 0.35–0.93; *p*-trend < 0.01); fruits and vegetables (hazard ratios 0.54; 95% confidence interval: 0.34–0.87 and 0.55; 95% confidence interval: 0.34–0.91, respectively). Findings from the cohort study showed that consumption of unprocessed and minimally processed foods was associated with a lower risk of CD. No significant associations were found in this cohort for UC patients [8]. However, in another European Prospective (EPIC) study, a diet with a high consumption of processed sugar and soft drinks alongside low consumption of vegetables was associated with a higher risk of UC [9]. In a recent case-control study in Iran, adherence to a “healthy” diet, categorized by low-fat dairy products, nuts, fish, dried fruits, vegetable oil, olive oil, fruits, and vegetables (all unprocessed), was protective against UC, whereas adherence to an “unhealthy” diet, which is high in processed meat, red meat, processed/high-fat dairy products, pickles, animal fats, potato chips, eggs, processed sugar, and processed desserts, correlated to an increased risk of UC [10].

In patients with a pre-existing IBD diagnosis, it has been found that ultra-processed-food consumption is reported as significantly higher (CD: Odds Ratio (OR) 1.94 (95% CI: 1.52–2.49, *p* < 0.001); UC: OR 1.39 (95% CI: 1.17–1.65, *p* < 0.001) compared to individuals without IBD. This higher intake of ultra-processed foods was also associated with an increased risk for IBD-related surgery (Hazard Ratio 4.06 (95% CI: 1.52–10.86, *p* = 0.005) [11]. In contrast, an intake of unprocessed white meat, unprocessed red meat, dairy, starchy foods, fruits, vegetables, and legumes was not associated with IBD risk. Since these foods were not found to be associated with the development of IBD, it is possible, according to Narula et al. [7], that the risk of developing IBD depends more on the way the food is processed (or ultra-processed) than the actual food itself [7]. However, future studies are needed to rule out confounding factors from the study. 

### 2.2. Emulsifiers in Inflammatory Bowel Disease

Emulsifiers have been shown, in recent studies, to play a role in microbiota dysbiosis and inflammatory responses that influence the development of IBD. Emulsifiers are food additives frequently used in food processing to extend shelf-life. However, some emulsifiers have pro-inflammatory effects [4].

The most studied emulsifiers are polysorbate 80 (P80) and carboxymethyl cellulose (CMC). CMC and P80 are commonly found in edible oils, ice creams, cake mixes, icing, and chocolate syrup, but ingestion of CMC and P80 emulsifiers are found to negatively impact intestinal microbiota [12]. Mice models investigating the consumption of these two emulsifiers, CMC and P80, showed exposure to these emulsifiers can induce microbiota penetration of the mucus layer that lines the intestinal mucosa. These microbiota alterations in mice led to chronic intestinal inflammation and “colitis in genetically susceptible hosts and as metabolic dysregulation in wild-type hosts” [4].

P80 administration caused similar alterations to human gut microbiomes as does IBD, which resulted in the reduction in beneficial Bifidobacterium and the reduction in important SCFA producers including Faecalibacterium and Subdoligranulum, as well as Clostridium leptum. In mice, an intake of P80 exacerbated ileitis, decreasing the α-diversity of intestinal microbiota. The growth of sulfide producers including Enterobacteriacaeae and the swarming behavior of the IBD-related species, Proteus mirabilis, were significantly increased in mice exposed to P80. This swarming behavior is a type of flagellar movement requiring multicellular processes and the differentiation of vegetative cells into specialized cells called swarmer cells. The promotion of swarming behavior in Proteus mirabilis is associated with its pathogenesis in IBD. Thus, in patients with IBD, intake of P80, as well as CMC, should be thoroughly considered due to the ability of these emulsifiers to destroy the mucosal barrier, promote colitis, and alter the gut microbiome populations and their functions [12].

In a controlled study of human subjects, the effects of CMC consumption were again observed to detrimentally alter the intestinal microbiome and fecal metabolome. These results emphasize the need for more studies focusing on the long-term effects of emulsifiers and the development of chronic inflammatory diseases in humans such as IBD [4]. Emulsifiers, thickeners, and other additives in many UPFs have been shown to disrupt and inflame the intestines; Lo et al., reported an experiment involving the administration of the synthetic emulsifiers, CMC and P80, in which the mucosal barrier was disrupted and the gut microbiome population was altered by inflammation, resulting in colitis [6].

In a recent study by Naimi et al. [13], 20 dietary emulsifiers were studied to show that P80 and CMC are not the only emulsifiers causing changes in the gut microbiota; the study found that carrageenans, gums, and sunflower lecithin can also lead to gut disruption of the microbiome [13]. A decrease in Clostridiales, specifically Faecalibacteria, was found after the intake of P80, iota carrageenan, and mono-diglycerides, while an increase in the Bacteriodales was associated with the consumption of the following emulsifiers: kappa carrageenan, lambda carrageenan, and glyceryl stearate. These findings were supported by the results of the study of Gerasimidis et al. [14] that showed an increase in Escherichia coli/Shigella after carrageenan kappa consumption and a reduction in Bifidobacterium after P80 and carrageenan-kappa consumption.

Depletion of Faecalibacterium and Bifidobacterium species with an increase in levels of *E. coli* are typically found in CD and UC patients. Studies by Naimi et al. [13] and Gerasimidis et al. [14] thus supported the hypothesis, stated in the review by Raoul et al. [15], that certain emulsifiers, including P80 and carrageenan-kappa, can exacerbate specific microbial variations that can be related back to CD and UC pathogenesis [15,16,17].

Sandall et al.’s murine study confirmed the inflammatory potential of various dietary emulsifiers by observing that a reduced colon length occurred following treatment with all the tested emulsifiers. Only P80 and CMC reached statistical significance, but gum arabic had highly variable effects through coordinate analysis. This study was the first to test commonly consumed emulsifiers, lecithin and gum arabic, for a gastrointestinal impact in mouse models. In Sandall et al.’s human study, it was found that soy lecithin and gum arabic were more commonly consumed than P80 and CMC in the diets of CD patients. Given that emulsifiers other than P80 and CMC approached significance for reducing colon length in human subjects, and consumption frequency of these other emulsifiers was high, an investigation testing the regulation of various emulsifier classes in the diets of CD patients should be undertaken [18].

As previously mentioned, the Naimi study reported findings that carrageenans are another emulsifier type that cause changes in the gut microbiota. Carrageenans are a group of sulfated polygalactans that are commonly found in flavored milks, iced coffee, dairy-based ice cream, and other frozen desserts. Carrageenan is directly metabolized by gut microbiota in the host which influences intestinal homeostasis. Liu et al., interestingly, noted that varying molecular weights of carrageenan have different effects. When carrageenan is divided into a low molecular weight, it increases intestinal permeability and leads to colitis onset. When it is of a high molecular weight, carrageenan may promote antitumor/antioxidant activities [12].

Carrageenan consumption has been reported to cause increases in Deferribacteres, Proteobacteria, Actinobacteria, Bacteroidetes, and Firmicutes. When carrageenan is of the κ-, ι-, and λ-isomers, it can induce harmful changes of α-diversity and increase microbiota inflammatory potential in the human gut. Bacteroides was promoted by λ-carrageenan while Faecalibacterium was decreased by ι-carrageenan. κ-carrageenan promoted Bacteroides and Shigella, decreased Bifidobacterium, and induced robust colitis in high-fat diet models [12]. Carrageenan in the κ-isomer form also drastically promoted two inflammation-related bacteria, Alistipes finegoldii and Bacteroides acidifacien [17]. In contrast, fecal bacteria diversity and abundance were enriched in rats when treated with ι-carrageenan [12].

A randomized controlled trial looked at 15 UC patients in remission to investigate whether a ‘no-carrageenan diet’ prevented relapse. All participants followed a diet free of carrageenan. The participants were then randomized to two arms: one arm would receive 200 mg/day of carrageenan in capsules and the other arm would receive a placebo dextrose tablet. After 1-year, three out of five patients in the carrageenan capsule group relapsed. In contrast, only one out of seven UC patients relapsed in the placebo, non-carrageenan diet group. The carrageenan capsule arm group experienced an increase in fecal calprotectin, although these results were not statistically significant. Fecal calprotectin remained stable in the non-carrageenan diet group [18]. This underscores the caution that UC patients may need to take to avoid carrageenan in their diet.

Glycerol monolaurate (GML) is an FDA-approved safe emulsifier that inhibits the growth of harmful bacteria, fungi, and viruses. GML is considered an antimicrobial-emulsifier. Prior work demonstrated that pre-treatment with GML is superior to co-treatment with GML for colitis; pre-treatment with GML increased the abundance of Lactobacillus and Bifidobacterium in feces and the levels of butyric and propionic acid, while also leading to a more rapid remission rate of colitis. This was measured by a reconstructed microbiome with an enhancement of fecal SCFAs. Furthermore, colitis remission induced by GML is associated with an altered gut microbiome, suggesting that it might be a friendly companion for IBD. However, more studies requiring long-term follow-up are necessary to test and qualify this assumption further [12].

## 3. Elimination and Exclusion Diets

### 3.1. Low FODMAP Diet

**Figure 2:**
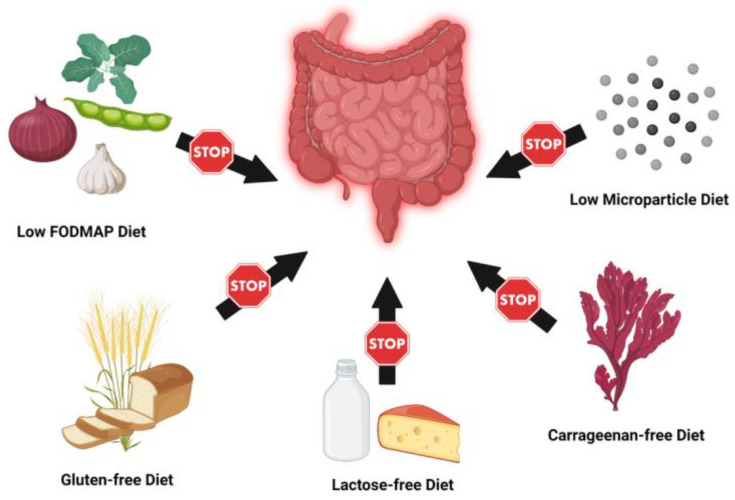
Elimination and Exclusion Diets in IBD.

FODMAP, which stands for fermentable, oligosaccharides, disaccharides, monosaccharides, and polyols, is a diet initially created for irritable bowel syndrome (IBS), which is enriched in short-chain carbohydrates that are poorly absorbed and highly fermented in the small intestine (Figure 2). This increased fermentation and poor absorption are hypothesized to lead to increased bloating, diarrhea, and abdominal pain. Consequently, a low FODMAP diet has gained popularity to help reduce these symptoms in patients with IBS and has been further trialed in many other gastrointestinal diseases, including IBD in attempts to find similar results. Multiple studies of varying sizes have shown significant decreases in IBS-like symptoms including bloating, abdominal pain, diarrhea, and overall symptom severity scores in patients with IBD consuming a low-FODMAP diet compared to a normal diet [19,20,21,22]. Low FODMAP groups also reported significantly increased rates of “normal” stool consistency [21]. Further, IBD patients on low FODMAP diets have shown higher general and IBD-specific quality of life scores, as well as rates of “satisfactory relief” after instituting the diet [19,20,21,22]. Of further support, IBD patients eating a high FODMAP diet reported an increased symptom burden [23]. In contrast, some studies have shown that the opposite may hold true. For example, IBD patients who are given a 3 d fructan (example of short-chain sugar) challenge showed increased pain, flatulence, and fecal urgency [24]. One hypothesized drawback to a low FODMAP diet in IBD is that the fecal samples of patients receiving a high FODMAP diet have been shown to increase rates of prebiotic bacteria [23]. The primary concern is that a low FODMAP diet may reduce symptoms but simultaneously reduce gut biodiversity and therefore overall gut health, leading to worse outcomes overall in IBD patients. Overall, current evidence suggests that low FODMAP diets can have beneficial effects in terms of symptom relief and increase patient satisfaction for IBD patients having IBS-like symptoms. However, the effects of a low FODMAP diet on IBD-specific pathology, IBD flares, severity, long-term sequelae, and response to treatment are yet to be determined.

### 3.2. Carrageenan-Free Diet

Another elimination diet undergoing research in patients with IBD is a carrageenan-free diet (Figure 2). Carrageenan is a food additive used as a thickening, stabilizing, texturizing, or emulsifying agent in a variety of foods, most commonly in dairy products and processed foods [25]. There have been multiple animal model studies demonstrating the correlation between carrageenan administration and increased rates of colitis [26]. These animal model studies are supported by in vitro experiments with human colonic tissue showing increased signs of inflammation with carrageenan exposure. Given these in vitro and in vivo animal studies, a pilot, randomized control trial of 12 UC patients in remission were all counseled to follow a no-carrageenan diet. They were then randomized to receive placebo capsules or carrageenan-containing capsules. The study showed that the “carrageenan-free” group had decreased rates of symptom relapse, as well as IL-6 and fecal calprotectin levels [27]. The results from the in vitro, in vivo animal models, and the small pilot study above indicate a possible correlation between decreased inflammation and a carrageenan-free diet. However, further large-sample, clinical studies in IBD patients are needed to elucidate the full effects of such a diet on that population.

### 3.3. Lactose-Free Diet

A lactose-free diet has been the mainstay treatment for GI symptoms in those with lactose intolerance for many years. Initial investigations of lactose intolerance in IBD patients (Figure 2) found similar rates in the IBD patient population compared to the general population, however, a recent meta-analysis found significantly increased rates of lactose intolerance in both CD and UC patients [28,29]. In terms of the lactose-free diet clinical effects, Gudman-Hoyerm et al. found that IBD patients with previously undiagnosed lactose-intolerance demonstrated significant improvement in their symptoms when changed to a lactose-free diet [28]. The study also trialed a lactose-free diet on 21 UC and 9 CD patients without lactose malabsorption. Of that group, five UC and three CD patients showed an improvement in symptoms, though no further statistical analysis was performed given the lack of a control group [28]. In contrast, a 2016 meta-analysis found that eating dairy products could even be protective against IBD [30]. The overall clinical impact of a lactose-free diet on IBD patients without diagnosed lactose intolerance remains unclear. The current literature indicates a benefit to testing and treating for lactose intolerance in IBD patients to relieve persistent symptoms despite disease control.

### 3.4. Gluten-Free Diet

Another ubiquitous elimination diet is a gluten-free diet. Classically, it was developed for the treatment of Celiac disease, which is an immune-mediated inflammatory disease of the small intestine due to sensitivity to dietary gluten. Given the similar inflammatory pathogenesis of Celiac disease and IBD, there has been much interest in the association between the two disease states. The studies to date have had contradicting results, and thus there is still controversy regarding the association between the two [31,32,33,34,35,36]. Regardless, many IBD patients have reported trialing a gluten-free diet when completing surveys for cross-sectional dietary studies (Figure 2). To date, there have been two large, internet-based studies assessing diets trialed, symptom improvement, and IBD flares. Of the 1647 patients surveyed in the United States, 314 (19.1%) reported having previously trialed a gluten-free diet, and 65.6% of patients described an improvement in their gastrointestinal symptoms, and 38.3% reported fewer or less severe IBD flares [37]. In the Switzerland cohort, 54 out of 1254 (4.7%) participants reported following a gluten-free diet. There were no significant differences in disease activity, hospitalization, or surgery. There were, however, significantly higher levels of anxiety and depression symptoms for those following a gluten-free diet [38]. Given the heterogeneity and different outcomes in these studies, as well as the lack of prospective studies, the effects of a gluten-free diet on IBD symptoms and flares remain elusive and require further evaluation.

### 3.5. Low Microparticle Diet

One of the earliest hallmarks of CD is the change in lymphoid aggregates in the gut known as Peyer’s Patches. Microparticles, which are defined as particles between 0.1 and 1.0 μm in size, have been shown to accumulate in phagocytes in this intestinal lymphoid tissue and are associated with inflammatory changes [39]. It has been hypothesized that microparticles with an actively charged surface strongly absorb luminal antigens, are brought into lymphoid tissue and phagocytes, and thus stimulate an aberrant immune response resulting in gut inflammation [40]. Studies have shown that after exposure to complexes of microparticles, intestinal cultures and biopsies of intestinal lamina propria have shown increased secretion of pro-inflammatory cytokines, particularly IL1-B. These findings lead to the notion that the ingestion of microparticles through diet can lead to increased inflammation in susceptible patients, such as those with IBD [41,42]. 

Microparticles are differentiated into two groups, endogenous and exogenous. Endogenous microparticles predominantly consist of calcium phosphate particles, whereas exogenous microparticles include both dietary contaminants such as dust or soil, and food additives such as TiO_2_ and aluminosilicates [40]. TiO_2_ is a whitening and food coloring additive commonly used in powdered foods, processed dairy products, sauces, toothpaste, and certain pharmaceuticals [43]. Aluminosalicates are frequently used in granular and powdered food products [43].

Recent studies have focused primarily on exogenous microparticles as they are commonly found at higher levels in Western diets and can theoretically be reduced with counseling (Figure 2). Lomer et al. conducted an initial double-blind pilot study of 18 total patients with ileal or ileal-colonic CD, randomized into either the microparticle diet counseling group or the control group without counseling. The CDAI (Crohn’s disease activity index) was significantly reduced in patients counseled on a low microparticle diet compared to controls [40]. However, a larger, follow-up study from the same group included 83 patients randomized to low/normal microparticle diet and/or low/normal calcium diet and found no significant difference in CDAI or remission rates after 16 weeks and 1 year [44]. The secondary analysis also found no difference in ESR, CRP, fecal calprotectin, or need for surgery in the low microparticle group compared to control groups [45].

Overall, the effect of a low microparticle diet on IBD remains inconclusive. While histological and in vitro studies suggest that microparticles lead to increased inflammatory cytokines and cell apoptosis in intestinal lymphoid tissue, data targeting clinical outcomes have yet to show benefit. Apart from the small pilot study by Lomer et al. [43], larger, prospective follow-up studies have shown no evidence of decreased clinical symptoms, changes in lab values, or remission rates in patients counseled on a low microparticle diet [44]. Further analysis should be completed on the relationship between calcium intake and its effects on gut inflammation as that may be a confounding factor for microparticle uptake and thus the induction of proinflammatory pathways. Regardless, there currently is insufficient evidence to counsel patients on a low microparticle diet with the goal of decreasing symptom burden or increasing remission rates.

The low FODMAP (fermentable, oligosaccharides, disaccharides, monosaccharides, and polyols) diet may reduce symptom burden and improve IBD-specific quality of life scores in patients with IBD. The gluten-free diet has been reported to reduce gastrointestinal symptoms and severity of flares in patients with IBD in cross-sectional studies, but there were no significant differences in disease activity, hospitalization, or surgery. The lactose-free diet is beneficial in patients with IBD with persistent symptoms despite disease control and undiagnosed lactose intolerance. Carrageenan is a food additive used as a thickening, stabilizing, texturizing, or emulsifying agent in a variety of foods, most commonly in dairy products and processed foods. A pilot randomized control trial of 12 UC patients in remission were all counseled to follow a no-carrageenan diet which resulted in decreased rates of symptom relapse, as well as IL-6 and fecal calprotectin levels. The low microparticle (0.1–1.0 uM) diet was shown to reduce CDAI in a small cohort of patients with CD. However, a larger follow-up study in CD found negative studies. The effects of the low microparticle diet in IBD remains inconclusive.

## 4. Specific Dietary Interventions

### 4.1. Mediterranean Diet

The Mediterranean diet (MD) is characterized by a high consumption of vegetables, fruits, nuts, legumes, unsaturated fats, and moderate consumption of fish and dairy; saturated fat, meat, and sweets are consumed sparingly [46]. In patients consuming a MD-like diet, fecal microbiome showed decreased Ruminococcus and Dorea, along with increased Faecalibacterium [47]. In patients with CD and UC, animal-derived foods are associated with increased Ruminococcus and elevated calprotectin [48].

**Figure 3:**
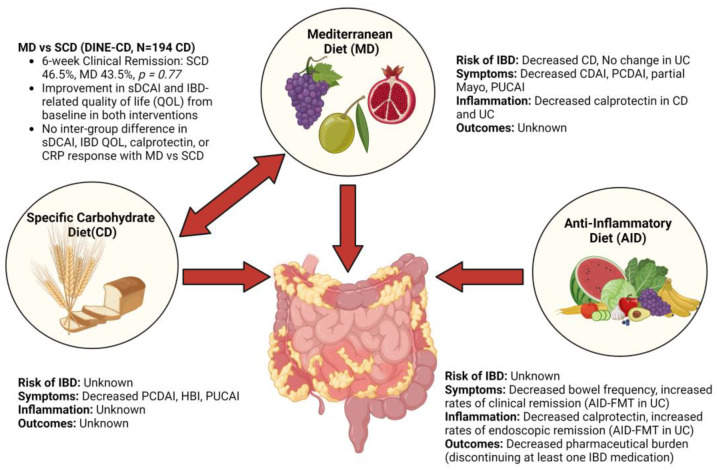
Specific Dietary Interventions in IBD: Mediterranean Diet (MD), Specific Carbohydrate Diet (SCD), and Anti-Inflammatory Diet (AID).

In previous studies, the MD was associated with a reduction in colorectal cancer, cardiovascular disease, and diabetes [49,50]. Given this, there has been considerable interest regarding the impact of the Mediterranean diet (MD) on IBD among patients and providers alike (Figure 3). In a study assessing attitudes towards the MD in patients with IBD, approximately 85% of patients believed that better dietary patterns could help control IBD symptoms, and a similar proportion was willing to visit a dietician to improve their diet [51].

Data regarding the role of the MD in the development of de novo IBD comes from a pooled analysis of two large national prospective cohort of Swedish patients, which showed that higher adherence to the MD was associated with a lower risk of CD among adults aged 45–79 years in a dose-dependent fashion (highest adherence compared to lowest, HR (hazard ratio) 0.42 [0.22–0.80], *P*trend = 0.02). The risk of UC was unchanged in patients with a high adherence to the MD compared to low adherence (HR 1.08 [0.74–1.58], *P*trend = 0.61) [52]. The impact of the MD on the development of IBD during the first epidemiologic peak of IBD remains unstudied.

Two small retrospective studies assessed the impact of the MD in adult patients with established IBD. In the first study, only nine patients fulfilled criteria for adherence, and all were men, greatly limiting conclusions [51]. A second study of 80 patients (62 CD, 18 UC) showed that there was a higher adherence to the MD among CD patients with inactive disease and those with active disease (43.2% vs. 9.3%, *p* < 0.001). There was no association for patients with UC [53]. A prospective study of 142 adult patients with IBD (58 CD, 84 UC) followed patients given the MD for 6 months. Disease activity was determined by Crohn’s disease activity index (CDAI) and partial Mayo score (PMS). Fewer patients with CD had active disease at 6 months compared to baseline (17.6% vs. 3.0%, *p* = 0.011) and there was a corresponding decline in a percentage of patients with a calprotectin over 250 (45.0% vs. 27.5%, *p* = 0.035). Similarly, in patients with UC there was an association with decreased disease activity (23.7% vs. 6.8%, *p* = 0.004) and elevated calprotectin (43.8% vs. 28.1%, *p* = 0.049)1. 

In a single center randomized trial, 100 pediatric patients with known mild-to-moderate IBD (ages 12 to 18) were assigned to the MD vs. usual diet for 12 weeks. Approximately 50 percent of both groups were prescribed either an aminosalicylate, immunomodulator, or biologic. There was a statistically significant decline in pediatric Crohn’s disease activity index (PCDAI: 6.4 ± 8.1 vs. 10.8 ± 7.4, *p* = 0.02) and pediatric ulcerative colitis activity index (PUCAI: 7.6 ± 11.2 vs. 9.2 ± 7.5, *p* = 0.04) at 12 weeks among patients assigned to the MD, but the clinical significance of this difference is unclear. Calprotectin was lower among patients who received the MD (221.5 ± 88.5 vs. 395.7 ± 194.4, *p* = 0.03) [54].

Data in post-surgical patients are limited to a single retrospective study of one hundred fifty patients with UC who previously underwent a colectomy and ileal pouch anal anastomosis (IPAA). Godney et al. showed that high adherence to a MD, in a multivariable model, was associated with decreased fecal calprotectin, but ultimately did not impact disease activity or pouchitis over 8 years of follow-up. In exploratory analysis, they identified high dietary fiber and dietary antioxidants as components of the MD that were associated with lower calprotectin [55].

### 4.2. Specific Carbohydrate Diet

There is considerable patient interest in the specific carbohydrate diet (SCD) as an adjunctive treatment for IBD. SCD specifies allowed and excluded foods; all fresh fruits and vegetables are acceptable except for starchy vegetables such as potatoes and yams. Unprocessed meats are allowed but processed or smoked meats are not. No grains are allowed. Honey is allowed as a sweetener, but processed sugars are not. Dairy is generally not allowed, but exception is made for certain cheeses [56].

Nutritional adequacy of dietary therapy in IBD is a key concern. A pilot study of eight patients with pediatric IBD suggested a SCD was adequate for growth and development. Six of eight (75%) patients gained weight on the diet. Mean energy intake relative to recommended daily allowance (RDA) was between 88%-145%. Macronutrient and micronutrient intake was largely adequate [57]. 

The efficacy of SCD (Figure 3) has been studied in pediatric patients, though studies uniformly have a small sample size. A single-center retrospective study of 26 pediatric patients (20 CD, 6 UC) showed decline in mean PCDAI from 14.5 ± 16.4 at baseline to 3.1 ± 5.1 at 6 months (*p* = 0.03); among the nine patients with active CD (PCDAI > 10), mean was 32.8 ± 13.2 at baseline and 8.8 ± 8.5 at 6 months. A similar decline was observed in PUCAI (six patients, two with active disease) [58]. A case series of seven pediatric patients with CD suggested improvement in symptoms and fecal calprotectin with a SCD [59]. Another case series of seven patients with CD showed that SCD resulted in symptomatic improvement in all patients, but none had normalization of fecal calprotectin or endoscopic mucosal healing [60]. 

A small prospective study followed nine patients with pediatric CD for 52 weeks. Compared to baseline values, at 12 weeks PDCAI (21.1 ± 5.9 to 7.8 ± 7.1, *p* = 0.011) declined, as did the Harvey–Bradshaw Index (HBI) and Lewis score (LS). At 52 weeks, the improvement in PDCAI (21.1 ± 5.9 to 5.4 ± 5.5, *p* = 0.016) and HBI was sustained; however, the improvement in LS was not sustained and only two of the seven patients had mucosal healing by capsule endoscopy [61]. A pilot study randomized 18 patients with only 10 patients completing the study (SCD = 4, modified SCD = 4, or whole food diet = 2). All patients had improvement in clinical symptoms at week 12. Microbiome, proteomics, and metabolomic analysis were performed, but a consistent signal was not obtained [62]. In a survey of 417 adult patients with IBD (70% female, mean age 34.9 years, 47% CD, 43% UC), 42% of patients reported symptomatic remission at 12 weeks compared to 4% at baseline.

### 4.3. Comparison of Mediterranean Diet and Specific Carbohydrate Diet

The efficacy of MD vs. SCD (Figure 3) was compared head-to-head in a randomized control trial of 194 patients with mild-to-moderate CD (Diet to Induce Remission in Crohn’s Disease: DINE-CD) [63]. Participants were randomized 1:1, stratified by whether the participant was currently using a biologic drug. Participants were recruited from 33 sites in the US and were followed for 12 weeks. Sixty three percent were female, ninety-one % were white. At screening, 57% were taking a biologic medication and 61% had non-stricturing, non-penetrating disease. The primary outcome was symptomatic remission at 6 weeks (short Crohn’s disease activity index—sCDAI <150), and key secondary outcomes were calprotectin response (<250 ug/g and reduction by >50% of for those with baseline calprotectin >250 ug/g) and c-reactive protein (CRP) response (<5 mg/L and reduction by >50% of for those with baseline CRP >5 mg/L). Participants were included if they had a diagnosis of CD and had a sCDAI between 175 and 400. Patients with an ostomy, stricture, or medication changes within a specific timeframe prior to study initiation were excluded. Analysis was completed with intention to treat.

The proportion of patients who achieve symptomatic remission at 6 weeks was not different between the two groups (SCD 46.5%, MD 43.5%, *p* = 0.77). Both groups had improvement in sCDAI and IBD-related quality of life compared to baseline but there was no difference between the two groups. Similarly, there was no inter-group difference in calprotectin or CRP response. Both diets were well-tolerated with low rates of serious adverse events in both groups. Microbiome alpha and beta diversity at baseline and at week 6 were not associated with symptomatic remission, and these values remained stable throughout the study. Both diets were moderately effective in mild-to-moderate CD, but SCD was not superior to the MD [63].

### 4.4. Ketogenic Diet

Many have heard of the Ketogenic diet (KD) in recent years due to its effectiveness for weight loss. With less than 50 g of carbohydrates per day, the KD alters the gut microbiota differently than a high-fat diet by elevating circulating ketone bodies, thus inhibiting the growth of Bifidobacterium and reducing the level of intestinal inflammatory Th17 cells [64].

**Figure 4:**
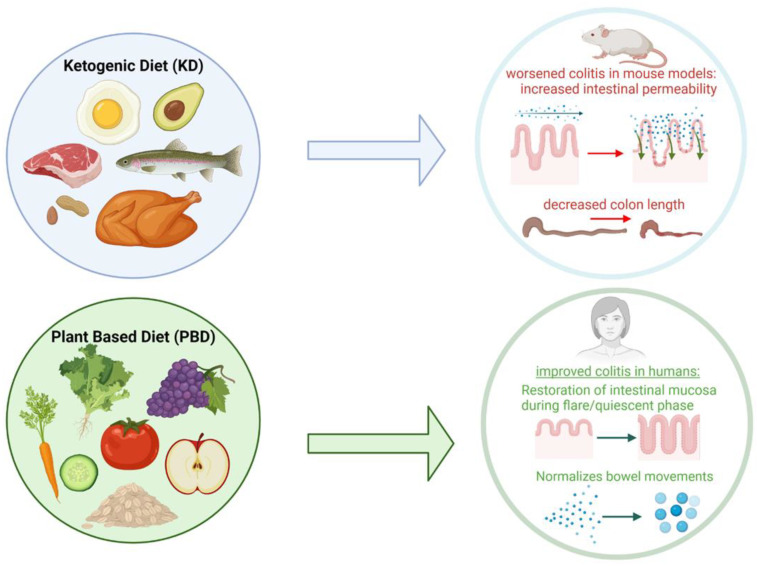
Specific Dietary Interventions in IBD: Ketogenic Diet (KD) and Plant-Based Diet (PBD) in IBD.

The KD would be beneficial to achieve ketosis, an anti-inflammatory state (Figure 4) characterized by increased levels of blood acetoacetate (ACA) and β-hydroxybutyrate (βOHB), the two main ketone bodies in the liver. 

The ketogenic diet has been shown to positively affect the gut of children by influencing the growth of commensal microbes producing short-chain fatty acids (SCFAs) (e.g., Bifidobacterium, Lactobacillus, Bacteroides, Faecalibacterium, Clostridium, and Ruminococcus). The anti-inflammatory effects of KD in reducing IBD in children may be due to an increase in anti-inflammatory βOHB made by SCFA-producing bacteria [65]. 

SCFA plays a key role in the relationship between KD and the gut microbiota of children with IBD. Butyrate and βOHB work in tandem to reduce intestinal inflammation, reduce inflammatory cytokine production, and increase histone acetylation in macrophages. Bacteria that produce SCFA play a large part in promoting gut barrier integrity and mitigating the dysbiosis in IBD colon mucosa. Since KD influences gut SCFA-producing bacteria in children, KD may be a possible approach to inducing remission and reducing inflammation in IBD [66]. However, future studies are needed to test if SCFA-producing gut bacteria are associated with specific inflammation markers after treatment with the KD.

A study by Kong et al. [66] study compared the gut microbiome and metabolic changes in mice fed with a KD, a low-carbohydrate diet (LCD), or a normal diet (ND). The effects of the KD on inflammation, gut microbiome, mucosal barriers, and immunity using a dextran sulfate sodium (DSS)-induced colitis model were used to highlight the beneficial effects of a KD-modified gut microbiome on intestinal inflammation using fecal microbiota transplantation (FMT) in a germ-free (GF) mouse model. Researchers found that the KD significantly reduced inflammatory responses, protected intestinal barrier function, reduced innate lymphoid cell (ILC3) production, and reduced expression of related inflammatory cytokines as compared to LCD [66]. While the Kong et al. study [66] suggested that a KD attenuated intestinal inflammation in a DSS murine colitis model, another study [67] showed the opposite findings. The Li et al. study [67] aimed at exploring the effects of a ketogenic diet on IBD in mice and its potential mechanisms; mouse models were given a KD or a control diet (CD) for a month and IBD was induced by diluting drinking water with 2% DSS during the last week of the diet. The study found that a KD actually worsened colitis by increasing intestinal permeability, decreasing expressions of intestinal epithelial barrier genes, increasing body weight loss, increasing DAI scores and histological scores, and decreasing colon length (a proxy measure of intestinal inflammation) [68]. The study’s mouse models indicated that KD increased intestinal injury through the following mechanisms; modification of intestinal microbes, metabolomic alterations of gut flora, destruction of the intestinal barrier, and exacerbation of inflammation in the blood and colon [67]. Furthermore, in other recent studies, keto diets were shown to “aggravate DSS-induced colitis with increased pathogenic bacteria such as Proteobacteria, Enterobacteriaceae, Helicobacter and Escherichia–Shigella and decreased potentially beneficial Erysipelotrichaceae” [68].

While ketogenic diets have worked as adjunct therapies to chemotherapy to reduce inflammatory cells, ketogenic diets have mostly “unfavorable” effects on the gut microbiome that require future research and caution [69]. Nonetheless, the true role that the KD has on IBD patients remains unknown and the long-term effects of the KD require further studies.

### 4.5. Plant-Based Diet

As developing countries move towards the Western diet of animal proteins, trans-fats, and processed sugars, increased cases of IBD are occurring, underscoring the link between a Western diet and IBD rates. In contrast, dietary fibers in heavily plant-based diets have been linked to a decreased risk of CD [70]. Data from a prospective cohort of 125,000 adults followed for over 30 years were used to correlate intake of a Western diet (prepared meals, processed beverages, red meat, and very few vegetables and fruits) to a higher risk of CD. This excessive intake of red/processed meat, processed sugars, and saturated fat is strongly linked to dysbiosis in the epithelial barrier function and the gut microbiome [71]. 

Plant-based foods have been shown to mitigate the effects of the Western diet by promoting the abundance of beneficial bacteria [70]. Plant-based diets (PBD) seem to aid in the restoration of the gut during an IBD flare and maintain gut symbiosis during a quiescent phase. While some experts had advised avoidance of dietary fiber during an IBD flare, other studies, specifically the 2019 Chiba et al. study [71], claimed this was not necessary; Crohn’s patients in an active flare phase, even in those with the stricturing CD, were given a plant-based diet (PBD) with high amounts of dietary fiber without problems [71]. 

PBDs are known to normalize bowel movements in constipated patients and in patients with diarrhea or loose stools (Figure 4) [71]. In the 2019 Chiba study of 46 past Crohn’s cases, infliximab combined with PBD induced a remission rate of 96%, an exciting finding considering that 30% of those suffering from IBD do not respond to infliximab. In patients who had their first episode of an UC flare, when treated with PBD as their therapy, there was only a 14% relapse rate after a year [72]. The researchers stated that treating mild cases of UC with PBD first, not medication, can induce remission in about one-third of patients with mild UC [71]. In the 2020 Chiba follow-up study, infliximab and a PBD as the first-line therapy induced remission in 13 of 17 severe UC patients, a remission rate of 76% [72]. Researchers noted that infliximab and PBD as first-line therapy provided better short-term and medium-term outcomes for patients with severe UC than outcomes reported in the past literature [72].

Many epidemiological studies have demonstrated an association between the Western diet, low fiber intake, and the development of colorectal cancer in the general population. Patients with IBD are at a higher risk of developing colorectal cancer compared to patients without IBD, thus, those with UC and CD eating a Western diet are at an even higher risk [48]. 

The gut microbiomes of individuals with colorectal cancer have a decreased amount of helpful anti-inflammatory microbes such as Clostridia spp., and an increase in inflammatory Fusobacterium, *Porphyromonas*, *Peptostreptococcus*, *Prevotella*, and mucosal *E. coli* [48]. Bolte et al. demonstrated that processed, animal-derived foods are associated with a higher abundance of the inflammatory species, *Ruminococcus*, as well as an elevated stool calprotectin value, a gut-specific inflammatory marker implicated in CD and UC [48].

### 4.6. Anti-Inflammatory Diet

Anti-inflammatory diets follow a similar framework to the specific carbohydrate diet, but rather than a focus on eliminating all gluten, AIDs have five main components which are intended to collectively mitigate the epithelial barrier dysfunction, microbial dysbiosis, and dysregulated immune responses often observed in IBD [73]. Regarding the specific foods, following AID includes limiting refined or processed complex carbohydrates which can disrupt the intraluminal gut flora by encouraging proliferation of pathogenic bacteria, increasing of pre- and probiotics, and modifying consumption of fatty acids to favor foods high in n-3-polyunsaturated fatty acids and avoid foods rich in n-6-polyunsaturated fatty acids [73,74,75]. Other key components of the AID are thorough review of individual diet patterns to identify nutritional gaps or food intolerances, and a stepwise adjustment in food texture by blending, grinding, or cooking to promote mucosal healing based on each patient’s symptoms [73,76]. The texture component is particularly unique to AID because it involves modification of not only the foods consumed but also how they are consumed. This allows for an increased intake of prebiotics without requiring patients to consume intact fiber, which can be incompatible with stricturing phenotypes. Conceptually, this type of diet is designed to support medical treatment of IBD (Figure 3) by limiting exposure to foods, additives, or components with the potential to contribute to proliferation of pathogenic microbes or otherwise promote inflammatory processes in the intestinal lumen and thereby mediate the mucosal immune response [73,74,75,76,77,78].

Subsequent studies have explored the use of AID in conjunction with medical treatment [73,77,78,79]. A small case study of 11 IBD patients (8 CD, 3 UC) who followed an anti-inflammatory diet (IBD-AID) for at least 4 weeks demonstrated the potential of this type of diet to influence clinical outcomes by reducing symptoms, disease severity, and the pharmaceutical burden [73]. IBD-AID was the first study to integrate adjustments to the food and textures of diet [73]. Patients following IBD-AID reported decreased bowel frequency and discontinuing at least one IBD medication [73]. However, these conclusions are limited by the size of the cohort and self-reported assessment dietary compliance. The highly individualized nature of this protocol limits the generalizability of the findings. Overall, this study has promising findings for a dietary intervention that emphasizes the intake of foods to optimize nutritional absorption and reduction in possibly inflammatory foods. The clinical findings suggest an improvement in dysbiosis, but since no samples were collected for comparison, any possible conclusions are based on prior studies which have drawn correlations between symptom improvement and improvement in dysbiosis and mucosal healing in various IBD patient populations [76]. 

A more recent study by the same group took a closer look at the mechanisms behind how anti-inflammatory diets can influence the microbiome and immune system of IBD patients to identify pathways to personalized nutritional recommendations approaches to treat dysbiosis in IBD patients using predictive analysis [77]. This study assessed possible changes in stool microbiome, metabolites, and serum cytokines of 19 patients (12 CD, 7 UC) before, during, and after following an anti-inflammatory diet for several weeks [77]. Comparison of stool microbiome and serum cytokine profiling pre- and post-intervention shotgun microbiome analyses from stool samples collected throughout the study period showed changes in microbiome, metabolomics, cytokines, and specific bacterial species abundance during the intervention window. Interestingly, increases in alpha and beta diversity of gut microbes were only noted during the intervention itself. Of the bacteria which increased abundance during the intervention, the 10 most abundant species belonged to SCFA-producing *Clostridia* and *Bacteroides* classes, which are often lower in abundance in IBD patients. Furthermore, the study was able to link consumption of certain prebiotic foods or adverse foods with an increase in protective (GM-CSF) and pro-inflammatory (IL-6, IL-8, and TNF-alpha) serum cytokines, respectively, which are major contributors to IBD inflammation and pathogenesis. However, the choice of study design used participants as their own control which cannot be overlooked as a limitation. Understanding how these dietary changes interact with microbiome and the immune responses in IBD patients to influence disease severity and progression would be interesting to explore in more depth in a more robust clinical trial. 

Other studies have investigated AID’s potential for prolonging remission and preventing subclinical colonic inflammation in adults with inactive UC, when compared to a diet consistent with Canadian national dietary recommendations (CFG) [78]. The study was unfortunately not well-powered enough to detect any significant difference in rates of clinical relapse between the two groups. After following the AID, patients saw a general decline in fecal calprotectin and had a significantly greater subclinical response (fecal calprotectin <150 µg/g at the endpoint) compared to patients following the CFG. Furthermore, the adherence to the AID appeared to maintain the downtrend of fecal calprotectin as higher fecal calprotectin levels were significantly correlated with eating fewer anti-inflammatory foods (i.e., yogurt, poultry, and seafood) and consuming more pro-inflammatory foods avoided in the AID (i.e., fruit juices, cured meat, and saturated fats). These findings strongly support the role of AID in delaying the onset of subclinical colitis and decreased likelihood of relapse. Patients following the AID also saw significant increases in the abundance of *Bifidobacteriaceae*, *Lachnospiraceae*, and *Ruminococcaceae* which were also significantly associated with lower fecal calprotectin levels. Significant changes in serum, urine, and stool metabolomes were also observed, particularly decreases in fecal acetone and xanthine levels as well as increases in serum pyruvic acid, and urinary p-hydroxybenzoic acid. The mechanistic conclusions from these findings show that the AID may play a role in prolonging remission and also demonstrated how the foods recommended by the AID may directly affect microbiome and metabolomic changes that decrease likelihood of clinical relapse amongst UC patients in remission.

Further evidence of direct microbiome manipulation in following an anti-inflammatory diet was found when an anti-inflammatory diet was combined with multi-donor FMT [79]. Patients with mild to moderately active UC following this protocol went into deep, sustained remission for a year following. After following the study-specific anti-inflammatory diet recommendations (FMT-AID) for 8 weeks vs. standard medical therapy (SMT), FMT-AID was superior to SMT in inducing clinical response (23/35 (65.7%) vs. 11/31 (35.5%), *p* = 0.01, OR 3.5 (95% CI 1.3 to 9.6)), remission (21/35 (60%) vs. 10/31 (32.3%), *p* = 0.02, OR 3.2 (95% CI 1.1 to 8.7)), and deep remission (12/33 (36.4%) vs. 2/23 (8.7%), *p* = 0.03, OR 6.0 (95% CI 1.2 to 30.2)) at 8 weeks. The anti-inflammatory diet was superior to SMT in maintaining deep remission until 48 weeks (6/24 (25%) vs. 0/27, *p* = 0.007). Patients in the FMT-AID group consumed less total fiber but the same amount of soluble fiber as the patients following standard medical therapy only. The combination of two strategies of manipulating the microbiome seemed to be especially powerful in inducing remission, but the findings suggested an anti-inflammatory diet could be key to sustaining steroid-free deep remission. 

Overall, the data from studies currently investigating the efficacy of anti-inflammatory diets in IBD suggest that this type of intervention may be promising at correcting symptoms and clinical outcomes attributed to dysbiosis. However, rigorous investigation into the anti-inflammatory diets as an intervention with robust clinical outcomes amongst patients with active disease is still needed. Anti-inflammatory diets could be a major foray into a shift in focus in IBD treatment from correcting dysregulated immune responses to correcting dysbiosis that precedes it.

### 4.7. High Fiber Diet

The anti-inflammatory effect facilitated by the conversion of dietary fiber to short-chain fatty acids (SCFA) by intestinal microbiota has been discussed at length by the recent literature [80,81]. In fact, the inflammation-mediating effects of SCFAs alongside indole compounds and dietary omega-3 polyunsaturated fatty acids has also been attributed to interactions between the gut microbiota and macrophages [82]. Further research has explored whether increasing consumption of dietary fiber in the diet could help mediate inflammation in patients with IBD. IBD mouse models have demonstrated how high-fat diets can exacerbate intestinal inflammation by interfering with the gut barrier function and the resident microbiome [82]. Investigation into possible clinical outcomes and changes in biomarkers in patients experiencing active IBD inflammation is an emerging area of research that several studies have begun to explore. 

In a study of 17 patients with UC either in remission or with very mild disease, a low fat, high fiber diet was noted to be associated with improved inflammatory markers and fewer signs of intestinal dysbiosis [83]. This was compared to similar, but less pronounced improvements in these same areas after patients followed a “healthier” version of the standard American diet for the same amount of time. Both diets included more fiber than the baseline diet of all participants prior to enrollment in the study, and notably patients reported improvements in patient quality of life (SIBDQ) from baseline following both interventions. A more profound decrease in inflammatory markers was identified following the low-fat, high fiber diet than the standard diet intervention. Furthermore, untargeted and targeted metabolomic studies on fecal samples collected following the low-fat, high fiber diet revealed significantly higher amounts of acetate than in samples collected at baseline. Stool samples collected from patients after the standard diet were also notable for higher acetate levels compared to baseline, but this increase was slightly less pronounced than that observed following the low-fat, high fiber diet. Fecal samples collected following the low-fat, high fiber diet were also notable for fewer signs of intestinal dysbiosis. In addition, microbiota composition was associated significantly with the amount of acetate and propionate found in the stool. When reflecting on the results of the study from a clinical standpoint, it is possible that this type of diet could benefit patients in remission from UC; however, because none of the patients entered the study during flare, and all patients stayed in remission throughout the study, no definitive conclusions can be drawn about the effect of the diet on inflammation during flare.

Previous reviews have discussed possible ways a high fiber diet might bolster intestinal barrier function, and the resulting decreased permeability would mediate the number of inflammatory triggers that can cause flare or systemic complications of IBD [45,84]. Whether or not a high fiber diet can be a good mediator of inflammation among patients with active disease, and what the impact is on disease activity and clinical laboratory findings would be an interesting future research direction.

### 4.8. Exclusive Enteral Nutrition (EEN)

Exclusive enteral nutrition (EEN) involves receiving all calories and nutritional requirements via liquid formulation while excluding all other food and drink except water. Enteral nutrition consists of mainly of amino acids and peptides. It is believed that limiting oral intake with EEN may reduce dietary antigens that may be triggers for CD [85]. The clinical benefits of EEN in CD have been explored in several studies. A prior meta-analysis of patients with CD on anti-TNF maintenance therapy [86] demonstrated that EEN was associated with greater odds of long-term remission. In a prospective study of 149 patients with CD with malnutrition, preoperative enteral nutrition support was associated with a trend but no conclusive evidence of a reduction in intra-abdominal septic complications [87]. On the contrary, a retrospective study of 300 patients with CD undergoing surgery demonstrated that preoperative oral enteral nutrition was well-tolerated and associated with a reduction in 30-day postoperative complications [88]. In another retrospective cohort study of CD patients, preoperative EEN was associated with downstaging the need for surgery in patients presenting with stricturing or penetrating complications, reduction in systemic inflammation, operative times, and incidence of post-operative abscess or anastomotic leak [89].

The Mediterranean diet (MD) is characterized by high consumption of vegetables, fruits, nuts, legumes, unsaturated fats, and moderate consumption of fish and dairy; saturated fat, meat, and sweets are consumed sparingly. Epidemiological studies suggest a decreased risk of CD with MD. Clinical trials of the MD in IBD have resulted in decreased symptoms, disease activity, and markers of inflammation. The specific carbohydrate diet (SCD) allows fresh fruits and vegetables except for starchy vegetables, unprocessed meats, honey, and certain cheeses are allowed, processed/smoked meats, processed sugars, and dairy are not allowed. SCD has been shown to decrease disease activity in IBD. Comparing MD vs. SCD (DINE-CD) showed similar rates for 6-weeek clinical remission and no difference between sCDAI, IBD-quality of life (QOL), CRP, and calprotectin. Anti-inflammatory diets (AID) follow a similar framework to the SCD, but rather than a focus on eliminating all gluten, AID includes limiting refined or processed complex carbohydrates, increasing of pre- and probiotics, and modifying the consumption of fatty acids to favor foods high in n-3-polyunsaturated fatty acids and avoidance of foods rich in n-6-polyunsaturated fatty acid. Other key components of the AID are thorough review of individual diet patterns to identify nutritional gaps or food intolerances, and a stepwise adjustment in food texture by blending, grinding, or cooking to promote mucosal healing based on each patient’s symptoms. AID has been associated with decreased bowel frequency and pharmaceutical burden in IBD. In a trial combined with FMT, AID-FMT lead to increased rates of clinical and endoscopic remission in UC.

KD has been shown to worsen colitis by increasing intestinal permeability, decreasing expressions of intestinal epithelial barrier genes, and decreasing colon length. PBDs are known to normalize bowel movements in constipated patients and in patients with diarrhea or loose stools. PBDs seem to aid in the restoration of the gut during an IBD flare and maintain gut symbiosis during a quiescent phase.

## 5. Emerging Dietary Interventions in IBD

### 5.1. Reduced Sulfur Diet in UC

In addition to the dietary interventions mentioned earlier in this review, several novel dietary approaches are being investigated in patients with IBD. One approach involves a reduced sulfur diet intervention in patients with UC (NCT04474561) [90]. This study is based on the premise that dysbiosis caused by higher levels of hydrogen sulfide and sulfate-reducing bacteria play a role in the pathogenesis of UC [91]. In this open label clinical trial, the effects of a reduced sulfur diet (includes reducing foods, food additives and beverages high in sulfate/sulfur, limiting sulfur-containing supplements, and consuming omega-3 fatty acid supplements to control overgrowth of sulfur-reducing bacteria in the colon) plus conventional management will be compared to conventional management for the primary outcome of disease activity measured by Mayo scores.

### 5.2. Personalized Fiber Diet in UC

While a high fiber diet may have some anti-inflammatory effects in animal models and small cohort studies of patients with IBD, the exact type and ideal amount of fiber to reduce inflammation and induce remission in patients with IBD is unknown. Armstrong et al. [92] recently demonstrated that unfermented dietary β-fructan fibers can induce proinflammatory cytokines in a subset of IBD intestinal biopsies cultured ex vivo, and immune cells. The results suggest that some dietary fibers have detrimental effects in select patients with active IBD who lack fermentative microbe activities. Based on these findings, the same group is currently investigating a personalized B-fructan diet in IBD patients (NCT05615779) [93]. In this dietary intervention, patients with UC or healthy controls will be randomized to consume foods high in pectin (placebo comparator), foods high in B-fructan, foods high in pectin based on personal host (biopsy) and microbe (stool) response at baseline, and foods high in B-fructan based on personal host (biopsy) and microbe (stool) response at baseline. The primary outcome of this study will be diet tolerability. Secondary outcomes include inflammatory response to diet (changes in markers of inflammation in blood and stool) and microbiota changes in response to diet.

### 5.3. Fermented Food Diet in UC

The effects of fermented foods on gastrointestinal inflammation and gut microbiome composition has been demonstrated in prior studies. Fermented plant extracts can attenuate dextran sodium sulfate-induced colitis in mice and production of proinflammatory cytokines. In addition, fermented plant extract supplementation in non-IBD subjects induced increases in Firmicutes phyla and in Clostridiales order, which play a central role in inflammation suppression [94]. Likewise, in a randomized, prospective study of healthy adult subjects, a high fermented-food diet steadily increased microbiota diversity and decreased inflammatory markers [95]. The effects of a fermented food-supplemented diet is being explored in an open label clinical trial in patients with UC (NCT04401605) [96]. In this trial, patients with active UC (elevated calprotectin and elevated symptom scores) will be assigned to an intervention of increasing the number of daily servings of fermented foods (e.g., Kimchi, Sauerkraut, Yoghurt, Kefir, etc.) over 10 weeks vs. control (regular diet). The primary outcome will be change in the clinical disease activity inflammatory marker, fecal calprotectin.

### 5.4. Caloric Restriction, Intermittent Fasting, Time Restricted Feeding Diets in IBD

Finally, novel directions in dietary interventions in IBD are extending beyond food composition and exploring the effects of caloric restriction and timing of food consumption. Intermittent fasting (IF) involves food consumption patterns in which individuals go extended time periods (e.g., 16–48 h) with little or no energy intake combined with intervening periods of normal food intake. Prior work has suggested that intermittent fasting may counteract age-related degenerative disease processes and may improve functional outcomes ranging from diabetes and cardiovascular disease to neurological disorders [97]. For example, a single fasting interval in humans can reduce basal concentrations of several metabolic biomarkers associated with chronic disease including insulin and glucose [98]. A prior mouse study of cycles of fasting and refeeding demonstrated that nutritional signals were critical in maintaining gut immune homeostasis [99]. In this study, temporary fasting drastically reduced the number of lymphocytes by 50% in Peyer’s patches with a large portion of germinal center and IgA + B cells lost during fasting. Furthermore, fasting may also abolish induction of antigen-specific IgA and oral tolerance [99]. 

Given the profound effects of intermittent fasting on the gastrointestinal immune response, clinical trials exploring the effects of intermittent fasting or fasting mimicking diets in IBD are underway. Two separate randomized, open-label clinical trials are currently investigating how an intermittent calorie-reduced diet (IRCD) that mimics fasting impacts inflammation in patients with mild to moderate UC (NCT03615690) [100] and CD (NCT04147585) [101]. In these clinical trials, three cycles of a 5-day reduced calorie diet will be compared with a regular diet control arm for the primary outcome of clinical response (decrease in partial Mayo score for UC, reduction in CDAI for CD). Secondary outcomes will also assess the effects of these dietary interventions on inflammatory biomarkers (CRP, ESR, calprotectin), endoscopic inflammation, quality of life indices, and immune cell and gut microbiome and metabolite composition. Likewise, another clinical trial will evaluate the impact of time-restricted feeding in Crohn’s disease (TRF-CD) (NCT04271748) [102]. In this open label clinical trial, patients with CD with active inflammation (defined by elevated CRP, calprotectin, or CD-SES) will be required to fast for 16 consecutive hours (e.g., 7:00 p.m.–11:00 a.m.) daily for 4 weeks. Subjects will be allowed to consume their normal diet during a chosen 8-h eating window. The primary outcomes for this study include change in Patient Recorded Outcome 2 (PRO2) scores and inflammatory markers (CRP and calprotectin). Secondary outcomes include change in gut microbiome composition and peripheral blood immune markers measured by mass cytometry (CYTOF).

## 6. Conclusions and Future Directions

The large number of observational and interventional studies evaluating the link between diet and IBD risk, pathogenesis, and role as a therapy in IBD underscores the significant interest and clinical need of patients and clinicians. Epidemiological studies have suggested that ultra-processed foods are associated with a higher incidence of IBD, whereas certain food additives and emulsifiers may disrupt gut microbiota homeostasis and lead to intestinal inflammation. Exclusion and elimination diets may be associated with improved symptoms in patients with IBD, but no definitive evidence exists for attenuating objective markers of inflammation or improving clinical outcomes. Specific dietary interventions such as the Mediterranean diet and the specific carbohydrate diet may decrease disease activity and calprotectin but are limited by small sample sizes. Similarly, dietary interventions such as high fiber, plant-based, ketogenic, and anti-inflammatory diets have all been shown to reduce symptoms, improve inflammatory burden, and quality of life metrics to varying degrees, but there exists significant heterogeneity in response to these dietary interventions. Exclusive enteral nutrition may be beneficial in a subset of pediatric CD patients and CD undergoing surgical interventions. 

Currently, most dietary studies in IBD are limited by study design, risk of bias and confounding, small sample size/underpowering, lack of controls and/or randomization, and significant heterogeneity in definitions for response and outcomes. Furthermore, most dietary trials have only assessed outcomes at short time-periods and the feasibility, tolerability, and long-term adherence of these dietary interventions remain to be determined. To date, there is no robust evidence that any dietary intervention alone may replace standard therapies in patients with IBD. However, diet may play an adjunct role to induce or maintain clinical remission with current standard IBD therapies. Novel approaches to dietary interventions in IBD such as personalized fiber, intermittent fasting, and time-restricted diets may provide clinical benefits and reveal innovative insights into IBD pathogenesis. The results of these ongoing dietary clinical trials in IBD are eagerly awaited. While no definitive recommendations regarding specific dietary interventions as therapy in IBD can be made at this time based on quality of current evidence, clinicians together with a dedicated nutritionist should discuss types of dietary interventions previously investigated, possible benefits and risks, and quality of evidence with patients who may be interested in incorporating dietary changes to their standard therapies. 

## Figures and Tables

**Table 1:** **Description of Diets Reviewed in the Article.** This article reviews distinct dietary patterns which have been studied in IBD. The table provides a description of the diet and provides examples of included and excluded foods.

Dietary Patterns	Description of Diet
Ultra-processed foods (UPF)	Ready-to-consume foods, typically created by industrial processes. Rich in additives (sweeteners, emulsifiers, flavors). Examples of UPFs include bacon, brownies, and soda.
FODMAP diet	Eliminates short-chain carbohydrates that are poorly absorbed in the small bowel (**F**ermentable **O**ligosaccharides, **D**isaccharides, **M**onosaccharides, and **P**olyols). Examples of FODMAPs include dairy, beans, cherries, and onions.
Carrageenan-free Diet	Eliminates carrageenan, a highly sulfated polysaccharide food additive commonly added to dairy products (ice cream, yogurt) and sandwich meats.
Gluten-free diet (GFD)	Eliminates gluten, a structural protein found in grains. Examples of gluten-containing foods include bread, beer, and soy sauce
Lactose-free diet	Eliminates lactose, a disaccharide protein found in dairy. Examples of lactose-containing foods include milk, cheese, and butter.
Low microparticle diet	Diet that eliminates additives with particle size <1 uM, such as titanium dioxide. Examples of microparticle-containing foods include powdered sugar and processed dairy.
Mediterranean diet (MD)	High consumption of olive oil, vegetables, fruits, nuts; moderate consumption of fish and dairy; limited meat and unsaturated fats.
Specific carbohydrate diet (SCD)	Allows non-starchy vegetables, fruits, unprocessed meats. No grains, processed meats or sugars, or starchy vegetables (such as yams).
Ketogenic diet	Low carbohydrate, high fat diet that induces ketosis. Favors meats, eggs, dairy, and non-starchy vegetables.
Plant-based diet	Diet that eliminates or minimizes non-plant-based foods; not necessarily entirely vegetarian or vegan.
Anti-inflammatory diet (AID)	Eliminates processed carbohydrates, increases n-3-polyunsaturated fatty acids, and decreases n-6-polyunsaturated fatty acids. Examples of disallowed foods includes cured meats, fruit juices, and many dairy items.
High fiber diet	Diet enriched with fruits, vegetables, legumes, and nuts. Generally above 30 g of fiber per day.
Exclusive enteral nutrition (EEN)	Nutrition exclusively through liquid formula. Only water is allowed beyond the formula-based diet.

## Data Availability

Not applicable.

## Supplementary Materials

The following supporting information can be downloaded at: https://www.mdpi.com/article/10.3390/nu15030579/s1, Table S1: A succinct summary of the included clinical dietary studies, study design, patient population, and clinical outcomes.

## References

[1] Kaplan G.G., Windsor J.W. (2021). The four epidemiological stages in the global evolution of inflammatory bowel disease. Nat. Rev. Gastroenterol. Hepatol..

[2] Shouval D.S., Rufo P.A. (2017). The Role of Environmental Factors in the Pathogenesis of Inflammatory Bowel Diseases: A Review. JAMA Pediatr..

[3] Cohen N.A., Rubin D.T. (2021). New targets in inflammatory bowel disease therapy: 2021. Curr. Opin. Gastroenterol..

[4] Srour B., Kordahi M.C., Bonazzi E., Deschasaux-Tanguy M., Touvier M., Chassaing B. (2022). Ultra-processed foods and human health: From epidemiological evidence to mechanistic insights. Lancet Gastroenterol. Hepatol..

[5] Teo K., Chow C.K., Vaz M., Rangarajan S., Yusuf S. (2009). PURE Investigators-Writing Group. The Prospective Urban Rural Epidemiology (PURE) study: Examining the impact of societal influences on chronic noncommunicable diseases in low-, middle-, and high-income countries. Am. Heart J..

[6] Lo C.-H., Khandpur N., Rossato S.L., Lochhead P., Lopes E.W., Burke K.E., Richter J.M., Song M., Ardisson Korat A.V., Sun Q. (2022). Ultra-processed Foods and Risk of Crohn’s Disease and Ulcerative Colitis: A Prospective Cohort Study. Clin. Gastroenterol. Hepatol..

[7] Narula N., Wong E.C.L., Dehghan M., Mente A., Rangarajan S., Lanas F., Lopez-Jaramillo P., Rohatgi P., Lakshmi P.V.M., Varma R.P. (2021). Association of ultra-processed food intake with risk of inflammatory bowel disease: Prospective cohort study. BMJ.

[8] Meyer A., Dong C., Casagrande C., Chan S., Huybrechts I., Nicolas G., Rauber F., Levy R.B., Millett C., Oldenburg B. (2022). Food processing and risk of Crohn’s disease and ulcerative colitis: A European Prospective Cohort Study. Clin. Gastroenterol. Hepatol..

[9] Vasseur P., Dugelay E., Benamouzig R., Savoye G., Lan A., Srour B., Hercberg S., Touvier M., Hugot J.-P., Julia C. (2021). Dietary Patterns, Ultra-processed Food, and the Risk of Inflammatory Bowel Diseases in the NutriNet-Santé Cohort. Inflamm. Bowel Dis..

[10] Rashvand S., Behrooz M., Samsamikor M., Jacobson K., Hekmatdoost A. (2018). Dietary patterns and risk of ulcerative colitis: A case-control study. J. Hum. Nutr. Diet. Off. J. Br. Diet. Assoc..

[11] Chen J., Wellens J., Kalla R., Fu T., Deng M., Zhang H., Yuan S., Wang X., Theodoratou E., Li X. (2022). Intake of ultra-processed foods is associated with an increased risk of Crohn’s disease: A cross-sectional and prospective analysis of 187,154 participants in the UK Biobank. J. Crohn’s Colitis.

[12] Liu C., Zhan S., Tian Z., Li N., Li T., Wu D., Zeng Z., Zhuang X. (2022). Food Additives Associated with Gut Microbiota Alterations in Inflammatory Bowel Disease: Friends or Enemies?. Nutrients.

[13] Naimi S., Viennois E., Gewirtz A.T., Chassaing B. (2021). Direct impact of commonly used dietary emulsifiers on human gut microbiota. Microbiome.

[14] Gerasimidis K., Bryden K., Chen X., Papachristou E., Verney A., Roig M., Hansen R., Nichols B., Papadopoulou R., Parrett A. (2020). The impact of food additives, artificial sweeteners and domestic hygiene products on the human gut microbiome and its fibre fermentation capacity. Eur. J. Nutr..

[15] Raoul P., Cintoni M., Palombaro M., Basso L., Rinninella E., Gasbarrini A., Mele M.C. (2022). Food Additives, a Key Environmental Factor in the Development of IBD through Gut Dysbiosis. Microorganisms.

[16] Sandall A.M., Cox S.R., Lindsay J.O., Gewirtz A.T., Chassaing B., Rossi M., Whelan K. (2020). Emulsifiers Impact Colonic Length in Mice and Emulsifier Restriction is Feasible in People with Crohn’s Disease. Nutrients.

[17] Mi Y., Chin Y.X., Cao W.X., Chang Y.G., Lim P.E., Xue C.H., Tang Q.J. (2020). Native κ-carrageenan induced-colitis is related to host intestinal microecology. Int. J. Biol. Macromol..

[18] Bancil A.S., Sandall A.M., Rossi M., Chassaing B., Lindsay J.O., Whelan K. (2021). Food Additive Emulsifiers and Their Impact on Gut Microbiome, Permeability, and Inflammation: Mechanistic Insights in Inflammatory Bowel Disease. J. Crohn’s Colitis.

[19] Cox S.R., Lindsay J.O., Fromentin S., Stagg A.J., McCarthy N.E., Galleron N., Ibraim S.B., Roume H., Levenez F., Pons N. (2020). Effects of Low FODMAP Diet on Symptoms, Fecal Microbiome, and Markers of Inflammation in Patients with Quiescent Inflammatory Bowel Disease in a Randomized Trial. Gastroenterology.

[20] Gearry R.B., Irving P.M., Barrett J.S., Nathan D.M., Shepherd S.J., Gibson P.R. (2009). Reduction of dietary poorly absorbed short-chain carbohydrates (FODMAPs) improves abdominal symptoms in patients with inflammatory bowel disease—A pilot study. J. Crohn’s Colitis.

[21] Prince A.C., Myers C.E., Joyce T., Irving P., Lomer M., Whelan K. (2016). Fermentable Carbohydrate Restriction (Low FODMAP Diet) in Clinical Practice Improves Functional Gastrointestinal Symptoms in Patients with Inflammatory Bowel Disease. Inflamm. Bowel Dis..

[22] Pedersen N., Ankersen D.V., Felding M., Wachmann H., Végh Z., Molzen L., Burisch J., Andersen J.R., Munkholm P. (2017). Low-FODMAP diet reduces irritable bowel symptoms in patients with inflammatory bowel disease. World J. Gastroenterol..

[23] Halmos E.P., Christophersen C.T., Bird A.R., Shepherd S.J., Muir J.G., Gibson P.R. (2016). Consistent Prebiotic Effect on Gut Microbiota with Altered FODMAP Intake in Patients with Crohn’s Disease: A Randomised, Controlled Cross-Over Trial of Well-Defined Diets. Clin. Transl. Gastroenterol..

[24] Cox S.R., Prince A.C., Myers C.E., Irving P.M., Lindsay J.O., Lomer M.C., Whelan K. (2017). Fermentable Carbohydrates [FODMAPs] Exacerbate Functional Gastrointestinal Symptoms in Patients with Inflammatory Bowel Disease: A Randomised, Double-Blind, Placebo-Controlled, Cross-over, Re-Challenge Trial. J. Crohn’s Colitis.

[25] Kakodkar S., Mutlu E.A. (2017). Diet as a Therapeutic Option for Adult Inflammatory Bowel Disease. Gastroenterol. Clin. N. Am..

[26] Tobacman J.K. (2001). Review of harmful gastrointestinal effects of carrageenan in animal experiments. Environ. Health Perspect..

[27] Bhattacharyya S., Shumard T., Xie H., Dodda A., Varady K.A., Feferman L., Halline A.G., Goldstein J.L., Hanauer S.B., Tobacman J.K. (2017). A randomized trial of the effects of the no-carrageenan diet on ulcerative colitis disease activity. Nutr. Healthy Aging.

[28] Gudmand-Hoyer E., Jarnum S. (1970). Incidence and clinical significance of lactose malabsorption in ulcerative colitis and Crohn’s disease. Gut.

[29] Asfari M.M., Sarmini M.T., Kendrick K., Hudgi A., Uy P., Sridhar S., Sifuentes H. (2020). Association between Inflammatory Bowel Disease and Lactose Intolerance: Fact or Fiction. Korean J. Gastroenterol..

[30] Szilagyi A., Galiatsatos P., Xue X. (2016). Systematic review and meta-analysis of lactose digestion, its impact on intolerance and nutritional effects of dairy food restriction in inflammatory bowel diseases. Nutr. J..

[31] Shah A., Walker M., Burger D., Martin N., Von Wulffen M., Koloski N., Jones M., Talley N.J., Holtmann G.J. (2019). Link Between Celiac Disease and Inflammatory Bowel Disease. J. Clin. Gastroenterol..

[32] Bar Yehuda S., Axlerod R., Toker O., Zigman N., Goren I., Mourad V., Lederman N., Cohen N., Matz E., Dushnitzky D. (2019). The Association of Inflammatory Bowel Diseases with Autoimmune Disorders: A Report from the epi-IIRN. J. Crohn’s Colitis.

[33] Casella G., D’Incà R., Oliva L., Daperno M., Saladino V., Zoli G., Annese V., Fries W., Cortellezzi C. (2010). Prevalence of celiac disease in inflammatory bowel diseases: An IG-IBD multicentre study. Dig. Liver Dis..

[34] Jandaghi E., Hojatnia M., Vahedi H., Shahbaz-Khani B., Kolahdoozan S., Ansari R. (2015). Is the Prevalence of Celiac Disease Higher than the General Population in Inflammatory Bowel Diseaese?. Middle East J. Dig. Dis..

[35] Leeds J.S., Höroldt B.S., Sidhu R., Hopper A.D., Robinson K., Toulson B., Dixon L., Lobo A.J., McAlindon M.E., Hurlstone D.P. (2007). Is there an association between coeliac disease and inflammatory bowel diseases? A study of relative prevalence in comparison with population controls. Scand. J. Gastroenterol..

[36] Limketkai B.N., Sepulveda R., Hing T., Shah N.D., Choe M., Limsui D., Shah S. (2018). Prevalence and factors associated with gluten sensitivity in inflammatory bowel disease. Scand. J. Gastroenterol..

[37] Herfarth H.H., Martin C.F., Sandler R.S., Kappelman M.D., Long M.D. (2014). Prevalence of a gluten-free diet and improvement of clinical symptoms in patients with inflammatory bowel diseases. Inflamm. Bowel Dis..

[38] Schreiner P., Yilmaz B., Rossel J.B., Franc Y., Misselwitz B., Scharl M., Zeitz J., Frei P., Greuter T., Vavricka S.R. (2019). Vegetarian or gluten-free diets in patients with inflammatory bowel disease are associated with lower psychological well-being and a different gut microbiota, but no beneficial effects on the course of the disease. United Eur. Gastroenterol. J..

[39] Powell J.J., Ainley C.C., Harvey R.S., Mason I.M., Kendall M.D., Sankey E.A., Dhillon A.P., Thompson R.P. (1996). Characterisation of inorganic microparticles in pigment cells of human gut associated lymphoid tissue. Gut.

[40] Lomer M.C.E., Thompson R.P.H., Powell J.J. (2002). Fine and ultrafine particles of the diet: Influence on the mucosal immune response and association with Crohn’s disease. Proc. Nutr. Soc..

[41] Ashwood P., Thompson R.P., Powell J.J. (2007). Fine particles that adsorb lipopolysaccharide via bridging calcium cations may mimic bacterial pathogenicity towards cells. Exp. Biol. Med..

[42] Evans S.M., Ashwood P., Warley A., Berisha F., Thompson R.P.H., Powell J.J. (2002). The role of dietary microparticles and calcium in apoptosis and interleukin-1beta release of intestinal macrophages. Gastroenterology.

[43] Lomer M.C., Harvey R.S., Evans S.M., Thompson R.P., Powell J.J. (2001). Efficacy and tolerability of a low microparticle diet in a double blind, randomized, pilot study in Crohn’s disease. Eur. J. Gastroenterol. Hepatol..

[44] Lomer M.C.E., Grainger S.L., Ede R., Catterall A.P., Greenfield S.M., Cowan R.E., Vicary F.R., Jenkins A.P., Fidler H., Harvey R.S. (2005). Lack of efficacy of a reduced microparticle diet in a multi-centred trial of patients with active Crohn’s disease. Eur. J. Gastroenterol. Hepatol..

[45] Limketkai B.N., Iheozor-Ejiofor Z., Gjuladin-Hellon T., Parian A., Matarese L.E., Bracewell K., MacDonald J.K., Gordon M., Mullin G.E. (2019). Dietary interventions for induction and maintenance of remission in inflammatory bowel disease. Cochrane Database Syst. Rev..

[46] Chicco F., Magrì S., Cingolani A., Paduano D., Pesenti M., Zara F., Tumbarello F., Urru E., Melis A., Casula L. (2021). Multidimensional Impact of Mediterranean Diet on IBD Patients. Inflamm. Bowel Dis..

[47] Turpin W., Dong M., Sasson G., Garay J.A., Espin-Garcia O., Lee S.H., Neustaeter A., Smith M.I., Leibovitzh H., Guttman D.S. (2022). Mediterranean-Like Dietary Pattern Associations with Gut Microbiome Composition and Subclinical Gastrointestinal Inflammation. Gastroenterology.

[48] Bolte L.A., Vila A.V., Imhann F., Collij V., Gacesa R., Peters V., Wijmenga C., Kurilshikov A., Campmans-Kuijpers M.J., Fu J. (2021). Long-term dietary patterns are associated with pro-inflammatory and anti-inflammatory features of the gut microbiome. Gut.

[49] Martínez-González M.A., Gea A., Ruiz-Canela M. (2019). The Mediterranean Diet and Cardiovascular Health. Circ. Res..

[50] Farinetti A., Zurlo V., Manenti A., Coppi F., Mattioli A.V. (2017). Mediterranean diet and colorectal cancer: A systematic review. Nutrition.

[51] Vrdoljak J., Vilović M., Živković P.M., Hadjina I.T., Rušić D., Bukić J., Borovac J.A., Božić J. (2020). Mediterranean Diet Adherence and Dietary Attitudes in Patients with Inflammatory Bowel Disease. Nutrients.

[52] Khalili H., Håkansson N., Chan S.S., Chen Y., Lochhead P., Ludvigsson J.F., Chan A.T., Hart A.R., Olén O., Wolk A. (2020). Adherence to a Mediterranean diet is associated with a lower risk of later-onset Crohn’s disease: Results from two large prospective cohort studies. Gut.

[53] Fiorindi C., Dinu M., Gavazzi E., Scaringi S., Ficari F., Nannoni A., Sofi F., Giudici F. (2021). Adherence to mediterranean diet in patients with inflammatory bowel disease. Clin. Nutr. ESPEN.

[54] El Amrousy D., Elashry H., Salamah A., Maher S., Abd-Elsalam S.M., Hasan S. (2022). Adherence to the Mediterranean Diet Improved Clinical Scores and Inflammatory Markers in Children with Active Inflammatory Bowel Disease: A Randomized Trial. J. Inflamm. Res..

[55] Godny L., Reshef L., Pfeffer-Gik T., Goren I., Yanai H., Tulchinsky H., Gophna U., Dotan I. (2020). Adherence to the Mediterranean diet is associated with decreased fecal calprotectin in patients with ulcerative colitis after pouch surgery. Eur. J. Nutr..

[56] Gottschall E. (1994). Breaking the Vicious Cycle: Intestinal Health through Diet.

[57] Braly K., Williamson N., Shaffer M.L., Lee D., Wahbeh G., Klein J., Giefer M., Suskind D.L. (2017). Nutritional Adequacy of the Specific Carbohydrate Diet in Pediatric Inflammatory Bowel Disease. J. Pediatr. Gastroenterol. Nutr..

[58] Obih C., Wahbeh G., Lee D., Braly K., Giefer M., Shaffer M.L., Nielson H., Suskind D.L. (2016). Specific carbohydrate diet for pediatric inflammatory bowel disease in clinical practice within an academic IBD center. Nutrition.

[59] Suskind D.L., Wahbeh G., Gregory N., Vendettuoli H., Christie D. (2014). Nutritional Therapy in Pediatric Crohn Disease: The Specific Carbohydrate Diet. J. Pediatr. Gastroenterol. Nutr..

[60] Wahbeh G.T., Ward B.T., Lee D.Y., Giefer M.J., Suskind D.L. (2017). Lack of Mucosal Healing from Modified Specific Carbohydrate Diet in Pediatric Patients with Crohn Disease. J. Pediatr. Gastroenterol. Nutr..

[61] Cohen S.A., Gold B.D., Oliva S., Lewis J., Stallworth A., Koch B., Eshee L., Mason D. (2014). Clinical and Mucosal Improvement with Specific Carbohydrate Diet in Pediatric Crohn Disease. J. Pediatr. Gastroenterol. Nutr..

[62] Suskind D.L., Lee D., Kim Y.M., Wahbeh G., Singh N., Braly K., Nuding M., Nicora C.D., Purvine S.O., Lipton M.S. (2020). The Specific Carbohydrate Diet and Diet Modification as Induction Therapy for Pediatric Crohn’s Disease: A Randomized Diet Controlled Trial. Nutrients.

[63] Lewis J.D., Sandler R., Brotherton C., Brensinger C., Li H., Kappelman M.D., Daniel S.G., Bittinger K., Albenberg L., Valentine J.F. (2021). A Randomized Trial Comparing the Specific Carbohydrate Diet to a Mediterranean Diet in Adults with Crohn’s Disease. Gastroenterology.

[64] Yan J., Wang L., Gu Y., Hou H., Liu T., Ding Y., Cao H. (2022). Dietary Patterns and Gut Microbiota Changes in Inflammatory Bowel Disease: Current Insights and Future Challenges. Nutrients.

[65] Alsharairi N.A. (2022). The Therapeutic Role of Short-Chain Fatty Acids Mediated Very Low-Calorie Ketogenic Diet–Gut Microbiota Relationships in Paediatric Inflammatory Bowel Diseases. Nutrients.

[66] Kong C., Yan X., Liu Y., Huang L., Zhu Y., He J., Gao R., Kalady M.F., Goel A., Qin H. (2021). Ketogenic diet alleviates colitis by reduction of colonic group 3 innate lymphoid cells through altering gut microbiome. Signal Transduct. Target. Ther..

[67] Li S., Zhuge A., Wang K., Lv L., Bian X., Yang L., Xia J., Jiang X., Wu W., Wang S. (2021). Ketogenic diet aggravates colitis, impairs intestinal barrier and alters gut microbiota and metabolism in DSS-induced mice. Food Funct..

[68] Trakman G.L., Fehily S., Basnayake C., Hamilton A.L., Russell E., Wilson-O’Brien A., Kamm M.A. (2022). Diet and gut microbiome in gastrointestinal disease. J. Gastroenterol. Hepatol..

[69] Tracy M., Khalili H. (2022). You Are What You Eat? Growing Evidence That Diet Influences the Risk of Inflammatory Bowel Disease. J. Crohn’s Colitis.

[70] Chiba M., Ishii H., Komatsu M. (2019). Recommendation of plant-based diets for inflammatory bowel disease. Transl. Pediatr..

[71] Chiba M., Nakane K., Tsuji T., Tsuda S., Ishii H., Ohno H., Watanabe K., Obara Y., Komatsu M., Sugawara T. (2019). Relapse Prevention by Plant-Based Diet Incorporated into Induction Therapy for Ulcerative Colitis: A Single-Group Trial. Perm. J..

[72] Chiba M., Tsuji T., Nakane K., Tsuda S., Ishii H., Ohno H., Obara Y., Komatsu M., Tozawa H. (2020). High Remission Rate with Infliximab and Plant-Based Diet as First-Line (IPF) Therapy for Severe Ulcerative Colitis: Single-Group Trial. Perm. J..

[73] Olendzki B.C., Silverstein T.D., Persuitte G.M., Ma Y., Baldwin K.R., Cave D. (2014). An anti-inflammatory diet as treatment for inflammatory bowel disease: A case series report. Nutr. J..

[74] Sheil B., Shanahan F., O’Mahony L. (2007). Probiotic effects on inflammatory bowel disease. J. Nutr..

[75] Schwiertz A., Jacobi M., Frick J.S., Richter M., Rusch K., Köhler H. (2010). Microbiota in pediatric inflammatory bowel disease. J. Pediatr..

[76] Osterman M.T. (2013). Mucosal healing in inflammatory bowel disease. J. Clin. Gastroenterol..

[77] Olendzki B., Bucci V., Cawley C., Maserati R., McManus M., Olednzki E., Madziar C., Chiang D., Ward D.V., Pellish R. (2022). Dietary manipulation of the gut microbiome in inflammatory bowel disease patients: Pilot study. Gut Microbes.

[78] Keshteli A.H., Valcheva R., Nickurak C., Park H., Mandal R., van Diepen K., Kroeker K.I., van Zanten S.V., Halloran B., Wishart D.S. (2022). Anti-Inflammatory Diet Prevents Subclinical Colonic Inflammation and Alters Metabolomic Profile of Ulcerative Colitis Patients in Clinical Remission. Nutrients.

[79] Kedia S., Virmani S., K Vuyyuru S., Kumar P., Kante B., Sahu P., Kaushal K., Farooqui M., Singh M., Verma M. (2022). Faecal microbiota transplantation with anti-inflammatory diet (FMT-AID) followed by anti-inflammatory diet alone is effective in inducing and maintaining remission over 1 year in mild to moderate ulcerative colitis: A randomised controlled trial. Gut.

[80] Galvez J., Rodríguez-Cabezas M.E., Zarzuelo A. (2005). Effects of dietary fiber on inflammatory bowel disease. Mol. Nutr. Food Res..

[81] Yusuf K., Saha S., Umar S. (2022). Health Benefits of Dietary Fiber for the Management of Inflammatory Bowel Disease. Biomedicines.

[82] O’Mahony C., Amamou A., Ghosh S. (2022). Diet-Microbiota Interplay: An Emerging Player in Macrophage Plasticity and Intestinal Health. Int. J. Mol. Sci..

[83] Fritsch J., Garces L., Quintero M.A., Pignac-Kobinger J., Santander A.M., Fernández I., Ban Y.J., Kwon D., Phillips M.C., Knight K. (2021). Low-Fat, High-Fiber Diet Reduces Markers of Inflammation and Dysbiosis and Improves Quality of Life in Patients with Ulcerative Colitis. Clin. Gastroenterol. Hepatol..

[84] Jiang Y., Jarr K., Layton C., Gardner C.D., Ashouri J.F., Abreu M.T., Sinha S.R. (2021). Therapeutic Implications of Diet in Inflammatory Bowel Disease and Related Immune-Mediated Inflammatory Diseases. Nutrients.

[85] Racine A., Carbonnel F., Chan S.S., Hart A.R., Bueno-de-Mesquita H.B., Oldenburg B., van Schaik F.D., Tjønneland A., Olsen A., Dahm C.C. (2016). Dietary Patterns and Risk of Inflammatory Bowel Disease in Europe: Results from the EPIC Study. Inflamm. Bowel Dis..

[86] Hirai F., Takeda T., Takada Y., Kishi M., Beppu T., Takatsu N., Miyaoka M., Hisabe T., Yao K., Ueki T. (2020). Efficacy of enteral nutrition in patients with Crohn’s disease on maintenance anti-TNF-alpha antibody therapy: A meta-analysis. J. Gastroenterol..

[87] Abdalla S., Benoist S., Maggiori L., Zerbib P., Lefevre J.H., Denost Q., Germain A., Cotte E., Beyer-Berjot L., Corte H. (2021). Impact of preoperative enteral nutritional support on postoperative outcome in patients with Crohn’s disease complicated by malnutrition: Results of a subgroup analysis of the nationwide cohort registry from the GETAID Chirurgie group. Colorectal. Dis..

[88] Meade S., Patel K.V., Luber R.P., O’Hanlon D., Caracostea A., Pavlidis P., Honap S., Anandarajah C., Griffin N., Zeki S. (2022). A retrospective cohort study: Pre-operative oral enteral nutritional optimisation for Crohn’s disease in a UK tertiary IBD centre. Aliment. Pharm. Ther..

[89] Heerasing N., Thompson B., Hendy P., Heap G.A., Walker G., Bethune R., Mansfield S., Calvert C., Kennedy N.A., Ahmad T. (2017). Exclusive enteral nutrition provides an effective bridge to safer interval elective surgery for adults with Crohn’s disease. Aliment. Pharm. Ther..

[90] Reduced Sulfur Diet in Ulcerative Colitis Patients (UCS). Identifier: NCT04474561. NCT04474561.

[91] Teigen L.M., Geng Z., Sadowsky M.J., Vaughn B.P., Hamilton M.J., Khoruts A. (2019). Dietary Factors in Sulfur Metabolism and Pathogenesis of Ulcerative Colitis. Nutrients.

[92] Armstrong H.K., Bording-Jorgensen M., Santer D.M., Zhang Z., Valcheva R., Rieger A.M., Sung-Ho Kim J., Dijk S.I., Mahmood R., Ogungbola O. (2022). Unfermented β-fructan Fibers Fuel Inflammation in Select Inflammatory Bowel Disease Patients. Gastroenterology.

[93] Personalized B-fructan Diet in Inflammatory Bowel Disease Patients. Identifier: NCT056157791. https://clinicaltrials.gov/ct2/show/NCT05615779.

[94] Sugimoto M., Watanabe T., Takaoka M., Suzuki K., Murakami T., Murakami N., Sumikawa S. (2021). Anti-Inflammatory Effect on Colitis and Modulation of Microbiota by Fermented Plant Extract Supplementation. Fermentation.

[95] Wastyk H.C., Fragiadakis G.K., Perelman D., Dahan D., Merrill B.D., Yu F.B., Topf M., Gonzalez C.G., Van Treuren W., Han S. (2021). Gut-microbiota-targeted diets modulate human immune status. Cell.

[96] Fermented Food-Supplemented Diet in Ulcerative Colitis. Identifier: NCT04401605. NCT04401605.

[97] Mattson M.P., Longo V.D., Harvie M. (2017). Impact of intermittent fasting on health and disease processes. Ageing Res. Rev..

[98] Patterson R.E., Sears D.D. (2017). Metabolic Effects of Intermittent Fasting. Annu. Rev. Nutr..

[99] Nagai M., Noguchi R., Takahashi D., Morikawa T., Koshida K., Komiyama S., Ishihara N., Yamada T., Kawamura Y.I., Muroi K. (2019). Fasting-Refeeding Impacts Immune Cell Dynamics and Mucosal Immune Responses. Cell.

[100] The Influence of a Fasting Mimicking Diet on Ulcerative Colitis. Identifier: NCT03615690. NCT03615690.

[101] Effects of an Intermittent Reduced Calorie Diet on Crohn’s Disease. Identifier: NCT04147585. NCT04147585.

[102] The Impact of Time Restricted Feeding in Crohn’s Disease (TRF-CD). Identifier: NCT04271748. NCT04271748.

